# Legacy forest structure increases bird diversity and abundance in aging young forests

**DOI:** 10.1002/ece3.5967

**Published:** 2020-01-28

**Authors:** Juliana Hanle, Marlyse C. Duguid, Mark S. Ashton

**Affiliations:** ^1^ School of Forestry and Environmental Studies Yale University New Haven CT USA

**Keywords:** early successional, ecological succession, irregular shelterwood, oak, retention forestry, shrub‐nesting birds, silviculture

## Abstract

Many studies have demonstrated the importance of early‐successional forest habitat for breeding bird abundance, composition, and diversity. However, very few studies directly link measures of bird diversity, composition and abundance to measures of forest composition, and structure and their dynamic change over early succession. This study examines the relationships between breeding bird community composition and forest structure in regenerating broadleaf forests of southern New England, USA, separating the influences of ecological succession from retained stand structure. We conducted bird point counts and vegetation surveys across a chronosequence of forest stands that originated between 2 and 24 years previously in shelterwood timber harvests, a silvicultural method of regenerating oak‐mixed broadleaf forests. We distinguish between vegetation variables that relate to condition of forest regeneration and those that reflect legacy stand structure. Using principal components analyses, we confirmed the distinction between regeneration and legacy vegetation variables. We ran regression analysis to test for relationships between bird community variables, including nesting and foraging functional guild abundances, and vegetation variables. We confirmed these relationships with hierarchical partitioning. Our results demonstrate that regenerating and legacy vegetation correlate with bird community variables across stand phases and that the strength with which they drive bird community composition changes with forest succession. While measures of regeneration condition explain bird abundance and diversity variables during late initiation, legacy stand structure explains them during stem exclusion. Canopy cover, ground‐story diversity, and canopy structure diversity are the most powerful and consistent explanatory variables. Our results suggest that leaving varied legacy stand structure to promote habitat heterogeneity in shelterwood harvests contributes to greater bird community diversity. Interestingly, this is particularly important during the structurally depauperate phase of stem exclusion of young regenerating forests.

## Graphical abstract

Our results demonstrate that regenerating and legacy vegetation correlate with bird community variables across stand phases and that the strength with which they drive bird community composition changes with forest succession. While regeneration explains bird variables during late initiation, legacy stand structure explains them during stem exclusion. Canopy cover, ground‐story diversity, and canopy structure diversity are the most powerful and consistent explanatory variables. Results suggest that leaving varied legacy stand structure to promote habitat heterogeneity in shelterwood timber harvests contributes to greater bird community diversity, particularly during the depauperate phase of stem exclusion.

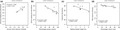

## 1 INTRODUCTION

Breeding bird communities reflect their habitat (Cody, 1985). Over the last century, dramatic changes of the age and structure of forests due to the suppression of disturbance regimes, agricultural abandonment, and declines in timber harvesting have drastically altered bird populations in eastern North America (Askins, 1998; Askins, Zuckerberg, & Novak, 2007; Brawn, Robinson, & Thompson, 2001; DeGraaf & Yamasaki, 2003; Litvaitis, 1993) and many temperate broadleaf forests worldwide (Block & Brennan, 1993; Gustafsson et al., 2012; MacArthur & MacArthur, 1961). Local breeding bird populations respond to regional influences, but are also very tightly tied to local habitat (Holmes & Sherry, 2001; Holmes, Sherry, & Sturges, 1986). The even‐aged, second‐growth temperate forests of North America, like those across much of Europe and Asia, become less structurally and compositionally diverse in the decades following forest stand initiation. As the density of developing forests increases, the canopy closes, and weaker trees die through self‐thinning (Oliver, Larson, & Oliver, 1996; Thomas & MacLellan, 2004); bird communities diminish and simplify as well (MacArthur & MacArthur, 1961). Harvesting to increase stand structural and species diversity can increase the abundance and diversity of bird species within these second‐growth forests in the initial decade following timber harvest (Fedrowitz et al., 2014; Gustafsson et al., 2012; James & Wamer, 1982; Johnston & Odum, 1956).

Timber harvests can restore shrubland and early‐successional regenerating forest (Ashton & Kelty, 2018). Over the last half century, northeastern North America has lost 85% of its early‐successional and old‐field habitat (Foster, Motzkin, & Slater, 1998). Shrublands provide critical habitat for shrub‐nesting bird species, many of whom are at risk or have seen sharp declines in their populations (DeGraaf & Yamasaki, 2003; King & Schlossberg, 2014; North American Bird Conservation Initiative US Committee, 2014; Schlossberg & King, 2007). These include species that are rare globally and continentally, such as the Golden‐winged Warbler (*Vermivora chrysoptera*) and the Prairie Warbler (*Setophaga discolor*) as well as species of regional concern, including the Blue‐winged Warbler (*Vermivora cyanoptera*), and those that are regionally common but still declining significantly, like the Chestnut‐sided Warbler (*Setophaga pensylvanica*) and the Eastern Towhee (*Pipilo erythrophthalmus*) (North American Bird Conservation Initiative US Committee, 2014; Schlossberg & King, 2007).

In the last decade, temperate second‐growth forest research has explored opportunities for forestry to maintain and increase early‐successional shrub habitats for wildlife (Vanderwel, Malcolm, & Mills, 2007). In particular, studies have demonstrated that regenerating shelterwoods host the greatest diversity and abundance of breeding birds in northeastern North American forests, attributing this to shelterwoods’ varied structure and composition providing habitat for multiple bird functional guilds (Ashton & Kelty, 2018; Duguid, Morrell, Goodale, & Ashton, 2016; Goodale, Lalbhai, Goodale, & Ashton, 2009; Keller, Richmond, & Smith, 2003; King & DeGraaf, 2000; Labbe & King, 2014; Perry & Thill, 2013). Shelterwood treatments, which coarsely imitate natural disturbances such as hurricanes, convectional windstorms, and tornadoes, include both early‐successional habitat and retained trees (Ashton & Kelty, 2018). Shelterwoods’ varying arrangements of retained legacy trees facilitate the regeneration of heavy‐seeded and poorly dispersed tree species that require partial shade for germination and seedling establishment, such as oaks, hickories, and maples (Ashton & Kelty, 2018). They also comprise an important silvicultural tool for managing for water conservation, carbon sequestration, and resilience to climate change (Ashton & Kelty, 2018). In irregular shelterwoods, which, in contrast to uniform shelterwood harvests, continue to contain legacy trees after a final tree removal, the legacy trees contribute to stand structural and age‐class diversity (Ashton & Kelty, 2018; Raymond, Bédard, Roy, Larouche, & Tremblay, 2009; Seidl, Rammer, & Spies, 2014). Recent research suggests that irregular shelterwoods may promote higher quality habitat for wildlife than uniform ones (Fedrowitz et al., 2014; Gustafsson et al., 2012).

Explanations for why the combination of early‐successional vegetation and legacy forest structure increases bird diversity and abundance have largely relied on proxy correlations and assumption. Some studies use indirect measures of forest structural change like time since harvest (Duguid et al., 2016; Goodale et al., 2009; Morris, Porneluzi, Haslerig, Clawson, & Faaborg, 2013. Others compare regeneration harvests, from shelterwoods to clear‐cuts, against original forest, forgoing measurement of vegetation over successional development (Keller et al., 2003; King & DeGraaf, 2000; Perry & Thill, 2013; Poulsen, 2002). Yet, forests fundamentally change with succession after disturbance, as described by the widely accepted stand dynamics model of forest succession (Oliver & Larson, 1990). Since postharvest changes in bird community composition with time have been attributed to regenerating vegetation but not statistically correlated, there remains a clear need to directly demonstrate relationships between changing forest structure and bird communities (Donner, Ribic, & Probst, 2010; Duguid et al., 2016; Thompson & DeGraaf, 2001).

Separating out the influences of ecological succession and retained stand structure for rich and abundant bird communities represents a challenge, but to do so provides a better understanding of how to create and manage irregular shelterwoods. Current management for birds may be based more on assumptions rather than data partly because of this analytical difficulty. To resolve it, we identified vegetation characteristics that reflected regeneration or retained structure. Using a combination of bird point counts and detailed vegetation surveys, we examine the following questions: (a) How does regenerating vegetation influence bird community composition and abundance in irregular shelterwood harvests over time? and (b) how does this differ from legacy vegetation influence on bird community composition and abundance?

## 2 METHODS

### 2.1 Study site

We conducted this study at Yale‐Myers Forest, a 3,213 ha research and demonstration forest in northeastern Connecticut (41°57′N, 72°07′W), a state in the eastern United States (Ashton, Duguid, Barrett, & Covey, 2015). The forest is actively and sustainably managed for timber. The topography is characterized by ridge and valley terrain ranging between 170 and 300 m amsl. Its soils are moderate to well‐drained glacial till soils. The climate is temperate‐humid, with temperatures that range from a July mean of 21.1°C to a January mean of 4.1°C and an annual rainfall that is approximately 120 cm/year.

The landscape is dominated by one‐hundred‐year‐old second‐growth oak‐broadleaf forest originating in the early 1900s from cut‐over old‐field white pine (*Pinus strobus*). Common midstory trees include black birch (*Betula lenta*), red maple (*Acer rubrum*), and sugar maple (*Acer saccharum*), while the canopy is dominated by several species of oaks (*Quercus velutina*, *Q. rubra*, *Q. alba*), white pine, and, more occasionally, hickory (*Carya* spp.) (Duguid, Frey, Ellum, Kelty, & Ashton, 2013; Frey, Ashton, McKenna, Ellum, & Finkral, 2007). Less commonly, eastern hemlock (*Tsuga canadensis*) and white pine dominate on sites of old woodlots and more recently abandoned fields, respectively. Irregular shelterwoods have been used as a regeneration method in Yale‐Myers Forest since the 1990s, and they retain legacy trees that range in number, basal area, species, and diameter, varying with site and prescription (Ashton et al., 2015).

Successional dynamics in Yale‐Myers Forest, which are consistent with observed change in other temperate mixed forests, have been documented in detail (Ashton & Kelty, 2018; Brunet et al., 2011; Donoso & Nyland, 2006; Duguid et al., 2016; Fredericksen et al., 1999; Oliver & Larson, 1990). Based on this research, and using the framework of the Oliver and Larson (1990) model of stand dynamics, we classified the stands included in our study by developmental phase. In early initiation (EI), the forest canopy remains largely open, while the groundcover, including tree seedlings, regenerates: The number of saplings increases, as does their height and average diameter at breast height (DBH). By late initiation (LI), the canopy has started to close and begins to exclude some herbs and shrubs. Within early stem exclusion (ESE), the canopy of the regenerating stand has fully closed, stem density decreases as saplings compete for sunlight, and the height and DBH of remaining stems increase. We included seven stands in early initiation (EI) regenerating from irregular shelterwood harvests cut 2–7 years previously, seven stands in late initiation (LI) regenerating from irregular shelterwood harvests cut 8–13 years previously, thirteen stands in early stem exclusion (ESE) regenerating from irregular shelterwood harvests cut 14–25 years previously, and eight closed‐canopied, unmanaged (UM) stands of the one‐hundred‐year‐old second‐growth forest.

### 2.2 Experimental design: bird surveys

We conducted bird surveys at 36‐point counts throughout Yale‐Myers Forest: 28 within regenerating shelterwood harvests and eight point counts in unmanaged stands. Each point was randomly placed within separate shelterwood and unmanaged stands, no closer than fifty meters from stand edges to minimize edge effect (Robbins, Sauer, & Droege, 1997; Taulman, 2013; Thompson, 2002). We sampled all stands in 2016.

We visited each point four times between late May and mid‐July, conducting eight counts a day between 5:30 and 10:00 a.m. in accordance with protocols used by Goodale et al. (2009) and Duguid et al. (2016). At each point, we waited at least one minute before beginning the point count. We recorded every bird heard or seen within a 50 m radius for 12 min. We did not conduct point counts on days with rain, or with winds greater than 24 kmph. We randomized the order in which we visited each group of eight point counts and changed the order in which we visited them so as to vary the time of day at which point count surveys were made.

### 2.3 Experimental design: vegetation surveys

Within three plots of radius 11.3 m (0.04 ha) located 19.4 m to the north, southwest, and southeast of the center of the bird point count plot, we recorded all trees >10 cm diameter at breast height (DBH), recording species, and canopy height class (understory, midstory, canopy, and emergent). We differentiated by height class between emergent trees, canopy trees (15–25 m), subcanopy trees (at least four meters below the canopy), and understory trees (at least 4 m below the subcanopy). We used a densitometer to measure percent canopy cover to the north, south, east, and west of the center of the plot and averaged the values for stand percent canopy cover.

To measure sapling regeneration, we laid six 4 × 4 m plots at 15.1 and 23.6 m away from each point count plot center on three transects (north, southeast, and southwest) in which we recorded the species, height, and diameter at breast height (DBH) of all saplings taller than 1.3 m and <10 cm DBH. To record ground‐story plant diversity and composition, we estimated percent cover forbs, ferns, and graminoids, woody debris, leaf litter, seedlings, and widespread and common shrub species that included the exotic invasives multiflora rose (*Rosa multiflora*), Japanese barberry (*Berberis thunbergii*); and the native shrubs blackberry (*Rubus allegheniensis*), mountain laurel (*Kalmia latifolia*), and witch hazel (*Hamamelis virginiana*).

### 2.4 Data analysis: birds

All analyses were done in R version 3.5.1 (R Core Team, 2018). We plotted species accumulation curves for each stand phase using 100 random permutations and, since the curves continued to rise, calculated Chao richness estimates for each stand using the vegan package (Oksanen et al., 2018).

We grouped birds by guilds, subsetting the data by life history traits, a tool used in ecological analysis and conservation planning (Bishop & Myers, 2005; Holmes, Bonney, & Pacala, 1979) (see Appendix S1). We classified observed species according to nest site and foraging strategy as listed by the Cornell Lab of Ornithology with three exceptions based on life history: blue‐winged warbler (*Vermivora cyanoptera*) as shrub‐nesting, hermit thrush (*Catharus guttatus*) and black‐throated blue warbler (*Setophaga caerulescens*) as forest ground‐nesting, and red‐winged blackbird (*Agelaius phoeniceus*) as marsh/open ground‐nesting. Foraging guilds do not correspond to nesting guilds.

We ran one‐way analysis of variance (ANOVA) on the following response variables (all normally distributed, as determined by the Shapiro–Wilk test with *p* > .05): (a) change in abundance of all birds; (b) bird species richness; (c) Chao richness estimate; (d) bird Shannon diversity index; (e) bird Shannon evenness index; (f) shrub‐nesting bird abundance; (g) forest ground‐nesting bird abundance; (h) tree‐nesting bird abundance; (i) cavity‐nesting bird abundance; (j) foliage‐gleaning bird abundance; and (k) ground‐foraging bird abundance, to test for differences across the three stages of stand development (EI, LI, and ESE) and the unmanaged forest. Additionally, we ran ANOVA and Tukey's post hoc analysis to test for differences across stand phase in species richness and Shannon evenness of the nesting and foraging guilds with normal distributions.

### 2.5 Data analysis: vegetation

Regenerating vegetation variables consist of percent canopy cover, median sapling height, ground‐story plant diversity, and total shrub cover. Legacy vegetation variables consist of stem density of trees of DBH >45 cm, legacy tree (>15 cm DBH) basal area, legacy tree stem density, total tree species richness, and the Shannon diversity of the canopy structure. The Shannon diversity of canopy structure categorized the number of stems in four height classes: understory, midstory, canopy, and emergent, as well as snags, using an application similar to the Shannon index applications of Kuuluvainen, Leinonen, Nygren, and Penttinen (1996), Staudhammer and LeMay (2001), and Man and Yang (2015).

While other studies have quantified canopy structure using measurements of canopy rugosity or crown area index, the amount of vegetative surface area relative to ground area, techniques made possible by intensive fieldwork or lidar, our approach can be accomplished by both ground surveys with canopy layer classification (Kane et al., 2011; Pretzsch & Schütze, 2008). We defined legacy trees as trees with DBH >15 cm based on field experience, knowing that (a) 15 cm DBH is approximately the low threshold for trees retained in a shelterwood harvest in this forest and (b) 15 cm DBH is larger than a sapling could possibly grow in the largest intervening period between harvest and data collection, 24 years.

To test for change in vegetation with phase of stand development (EI, LI, and ESE), we ran nonparametric Kruskal–Wallis tests on all non‐normally distributed vegetation variables and ANOVA on all normally distributed vegetation variables. To test our hypothesis that, across all stands, regenerating vegetation variables collectively represent one important influence of bird diversity and abundance, and legacy vegetation variables another, we ran principal components analysis on the correlation matrix of all vegetation variables. Additionally, to understand the relationships between canopy structure Shannon diversity and the other vegetation variables, we ran linear regression analyses on all positive canopy structure values that were normally distributed, as determined by the Shapiro–Wilk test. We also ran binomial regressions on measures of canopy structure diversity with all other vegetation variables.

### 2.6 Data analysis: birds and vegetation combined

We ran linear regression analysis to examine the separate effects of regenerating vegetation and legacy vegetation variables on bird community variables across and within the three stand phases. For relationships across all stand phases that showed evidence of modality, we subsequently ran quadratic regressions and report those correlations where the *r* ^2^ value was improved by more than .03. We chose simple regression and not multiple regression in order to isolate specific relationships between vegetation and bird variables and to avoid effects of collinearity or bias produced by techniques to circumvent collinearity (Smith, Koper, Francis, & Fahrig, 2009). We also ran simple regressions on individual bird species responses to vegetation variables across and within the three stand phases for birds with normal distributions.

We ran hierarchical partitioning to confirm our identification of predictor variables with the greatest explanatory power in relation to response variables and to check for type II error in our regression conclusions (Mac Nally, 1996, 2002). All analyses were run in RStudio version 0.99.903 (RStudio Team, 2016).

## 3 RESULTS

**Figure 1:**
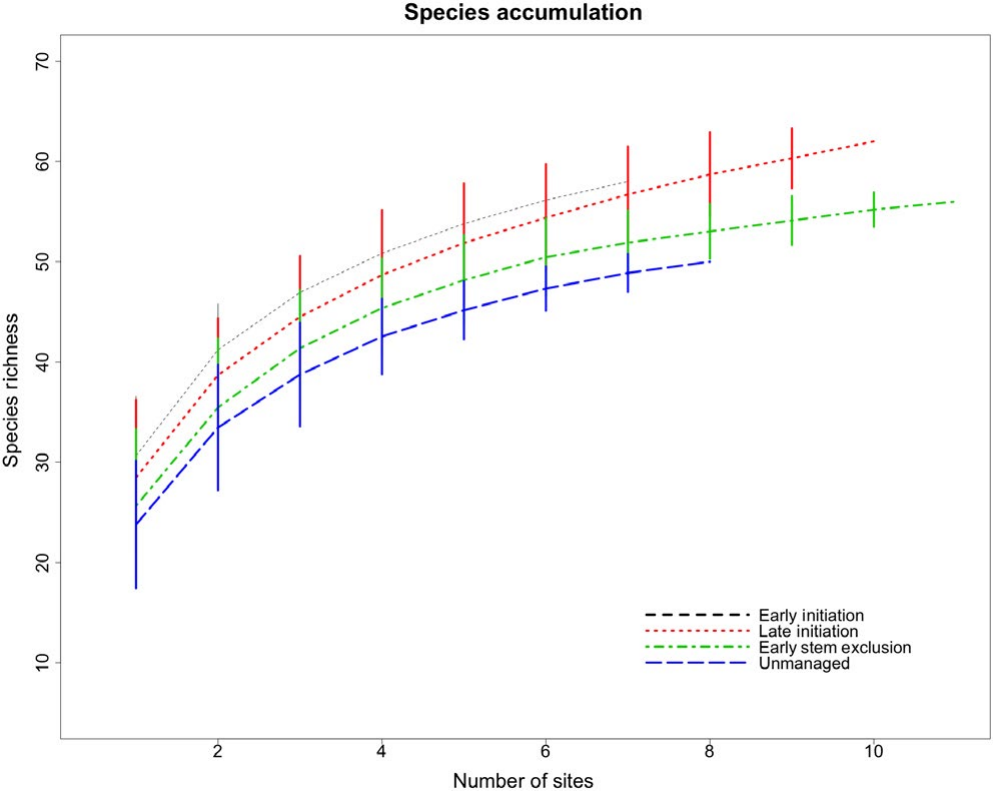
Species accumulation curves. Rarefaction by stand phase with random permutations 100 times

**Figure 2:**
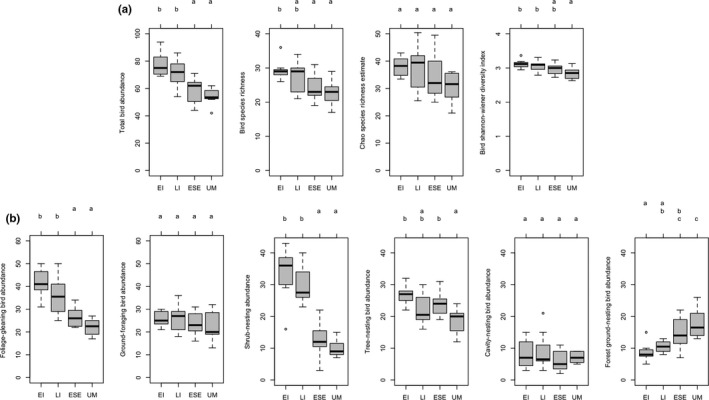
(a) Abundance, species richness, Chao richness estimate, and Shannon diversity of birds across stand phases (EI, early initiation; ESE, early stem exclusion; LI, late initiation; UM, control mature forest). (b) Abundance of birds in six nesting guilds across stand development phases. Letters denote differences (a < b < c) among stand phases using Tukey's post hoc analysis. Nesting‐type guilds—shrub‐nesting, tree canopy‐nesting, cavity‐nesting, and forest ground‐nesting species; foraging‐type guilds—foliage‐gleaning, and ground‐foraging

We detected 2,641 individual birds comprising 71 species across the 36 stands. We observed 57 species in early initiation (EI) stands, 57 species in late initiation (LI) stands, 62 species in early stem exclusion (ESE) stands, and 53 species in unmanaged stands (UM). Species accumulation curves were highest for EI, and then LI, ESE, and lastly UM stands (Figure 1). Overall abundance, species richness, and Shannon diversity decreased significantly between early initiation and unmanaged stands (Figure 2a), as it did in the data collected in 2014 (Duguid et al., 2016).

Shrub‐nesting, tree‐nesting, and foliage‐gleaning bird abundances significantly decreased with stand development phase and ground‐nesting bird abundance significantly increased (Figure 2b). Most of the loss of total abundance and species richness with stand phase can be attributed to changes in shrub‐nesting and foliage‐gleaning bird abundances (Figure 2b). By early stem exclusion (ESE), forest ground‐nesting birds were at the same abundance as unmanaged forest.

Within functional guilds, shrub‐nesting bird species richness and foliage‐gleaning species richness decreased significantly with stand phase (*F* = 6.63, *p* < .005; *F* = 5.92, *p* < .01). Nesting and foraging guild Shannon evenness was high, generally between 0.8 and 1 for all guilds, and did not change significantly with stand phase. This suggests that no abundance of a single or a small number of species drove guild abundance. Within stands in early initiation (EI), we observed shrub‐nesting bird species in need of conservation such as occasionally observing the prairie warbler (0.07 ± 0.12 observation frequency) and the blue‐winged warbler (0.07 ± 0.19), and regularly observing the chestnut‐sided warbler (1.46 ± 0.99), and the eastern towhee (1.82 ± 0.55). Other birds very closely associated with shrub habitat that we observed include common yellowthroat (*Geothlypis trichas*) (1.57 ± 0.35), gray catbird (*Dumetella carolinensis*) (1.43 ± 0.85), yellow warbler (*Setophaga petechil*) (0.79 ± 0.71), and American goldfinch (*Spinus tristis*) (0.5 ± 0.32) (North American Bird Conservation Initiative US Committee, 2014; Schlossberg & King, 2007).

Principal components analysis resulted in legacy vegetation variables almost entirely comprising the first component and regenerating vegetation variables the second, which together accounted for 66% of the proportion of variance within vegetation (Component 1 proportion of variance = 0.48, Component 2 proportion of variance = 0.19). These results confirmed that regenerating and legacy vegetation variables separately capture two distinct underlying influences on bird communities (see Appendix S2). We excluded percent canopy cover from the analysis because of the appearance of nonlinear relationships with the other vegetation variables.

### 3.1 Regeneration effect

The regenerating vegetation variables, percent canopy cover, sapling median height, ground‐story plant Shannon diversity, and total shrub cover, changed significantly with stand phase (*H* = 15.3, *p* < .0005; *H* = 8.11, *p* < .02; *F* = 8.58, *p* < .002; *H* = 8.9, *p* < .012, respectively), supporting our assumption that they reflect forest succession.

**Figure 3:**
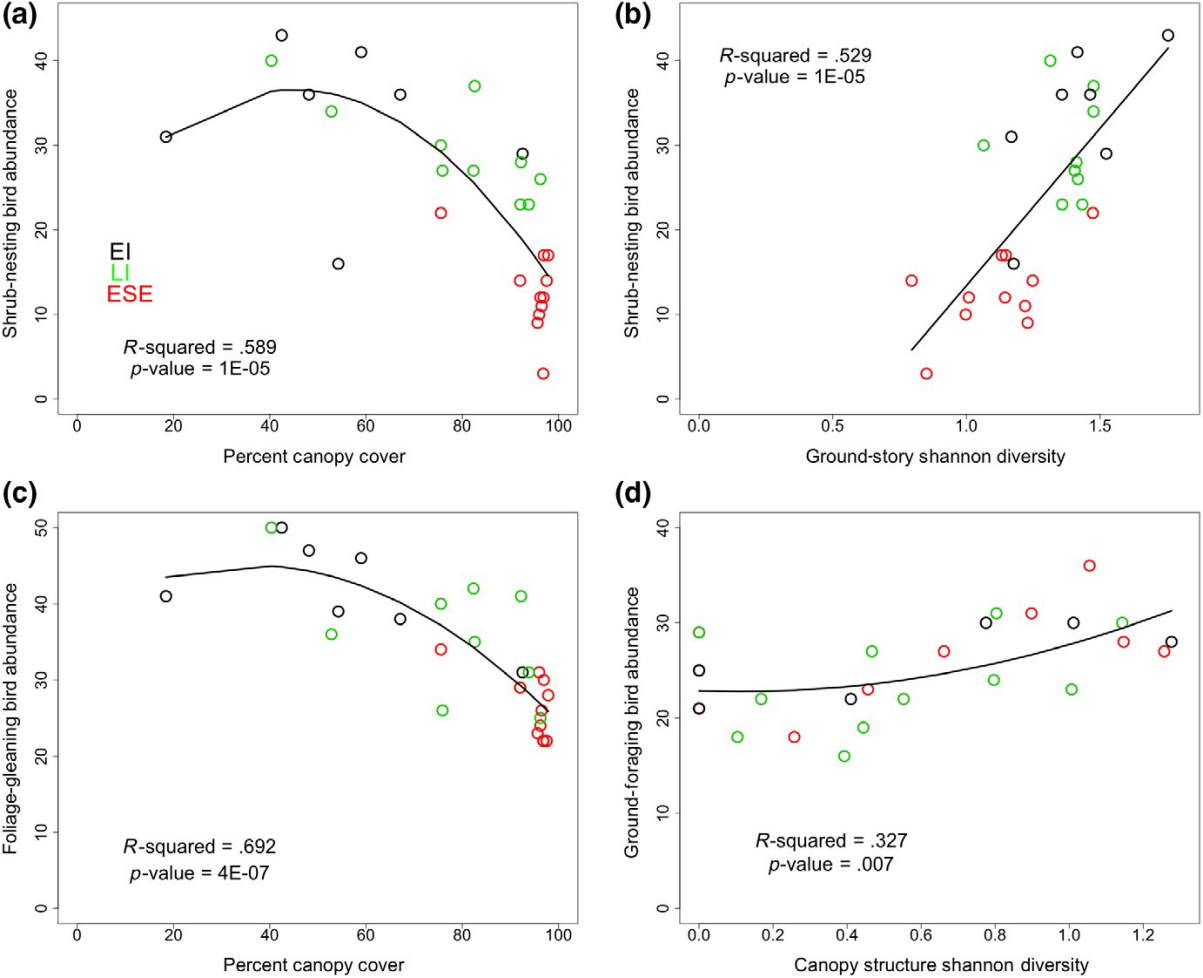
Regressions depict (a) shrub‐nesting bird abundance against percent canopy cover, (b) shrub‐nesting bird abundance against ground‐story plant Shannon diversity, (c) foliage‐gleaning bird abundance against percent canopy cover, and (d) ground‐foraging bird abundance against canopy structure diversity. Data from stands in early initiation (EI), late initiation (LI), and early stem exclusion (ESE) are colored black, green, and red, respectively

Across all stand phases, increasing canopy cover negatively correlates with total bird abundance, bird species richness, shrub‐nesting bird abundance, and foliage‐gleaning bird abundance (Table 1; Figure 3) and positively correlates with forest ground‐nesting bird abundance (Table 1). For total bird abundance, shrub‐nesting bird abundance, and foliage‐gleaning bird abundance, this relationship is best captured by quadratic regressions. Within late initiation (LI), canopy cover negatively correlates with shrub‐nesting bird abundance and tree‐nesting bird abundance (Table 1).

**Table 1:** Effects of regenerating stand structure on bird communities across stand phases and within late stand initiation. Significant correlations in bold. Positive correlations are denoted by plus signs in dark shading and negative correlations by minus signs in light shading. Where the model is quadratic, an “I” value is present. For these models, the degrees of freedom are 2 and 25

(a) All											
	Percent canopy cover – 18%–98%						Sapling median height – 0–5 m				
		*I*	β	*F*_1,26_	*r* ^2^	*p*		β	*F*_1,26_	*r* ^2^	*p*
Total bird abundance	−	**−0.007**	**0.653**	**11.6**	**.48**	**3E‐04**	−	**−6.521**	**11.22**	**.301**	**.002**
Bird species richness	−		**−0.085**	**6.37**	**.20**	**.018**	−	**−2.111**	**8.90**	**.255**	**.006**
Chao richness estimator			−0.042	0.40	.01	.532		−2.358	2.89	.100	.101
Shannon diversity			−0.003	3.85	.13	.060	−	**−0.062**	**4.60**	**.150**	**.041**
Shannon evenness			0.000	0.90	.03	.350		0.004	1.85	.066	.185
Shrub‐nesting bird abundance	−	**−0.008**	**0.711**	**17.93**	**.59**	**1E‐05**	−	**−4.927**	**6.86**	**.209**	**.014**
Forest ground‐nesting bird abundance	+		**0.094**	**8.10**	**.24**	.008		0.947	1.41	.051	.246
Tree‐nesting bird abundance			−0.072	4.07	.13	.054		−0.796	0.95	.035	.337
Foliage‐gleaning bird abundance	−	**−0.005**	**0.360**	**28.12**	**.69**	**4E‐07**	−	**−3.880**	**6.83**	**.208**	**.015**
Ground‐foraging bird abundance			0.019	0.20	.01	.660		−1.586	3.33	.114	.079

	Shrub cover – 0%–82%						Ground cover diversity – 0.80–1.75				
		*I*	β	*F*_1,26_	*r* ^2^	*p*		β	*F*_1,26_	*r* ^2^	*p*
Total bird abundance	±	**−0.008**	**0.734**	**3.77**	**.23**	**.037**	+	**32.967**	**13.53**	**.342**	**.001**
Bird species richness	±	**−0.003**	**0.213**	**3.76**	**.23**	**.037**	+	**11.484**	**13.13**	**.335**	**.001**
Chao richness estimator			−0.007	0.019	.00	.892	+	**14.643**	**5.39**	**.172**	**.028**
Shannon diversity	±	**‐1E‐4**	**0.008**	**5.98**	**.32**	**.007**	+	**0.421**	**11.80**	**.312**	**.002**
Shannon evenness	−		**‐3E‐04**	**6.54**	**.20**	**.017**		0.005	0.15	.006	.705
Shrub‐nesting bird abundance	±	**−0.008**	**0.763**	**8.65**	**.41**	**.001**	+	**37.200**	**29.23**	**.529**	**1E‐05**
Forest ground‐nesting bird abundance			−0.037	1.54	.06	.225	−	**−11.590**	**13.48**	**.341**	**.001**
Tree‐nesting bird abundance			−0.040	1.83	.06	.187		1.664	0.18	.007	.675
Foliage‐gleaning bird abundance			0.093	1.92	.07	.178	+	**19.353**	**7.77**	**.230**	**.010**
Ground‐foraging bird abundance			0.016	0.23	.01	.644		8.339	4.21	.139	.051

(b) Late initiation										
	Percent canopy cover – 18%–98%					Median height – 0–3.5 m				
		β	*F*_1,26_	*r* ^2^	*p*		β	*F*_1,26_	*r* ^2^	*p*
Total bird abundance		−0.263	2.86	.26	.129	−	**−6.270**	**6.08**	**.43**	**.039**
Bird species richness		−0.032	0.16	.02	.696		−2.090	2.51	.24	.151
Chao richness estimator		0.033	0.04	.01	.848		−3.067	1.09	.12	.326
Shannon diversity		‐8E‐04	0.06	.01	.812		−0.052	0.85	.10	.383
Shannon evenness		1E‐04	0.19	.02	.676		0.007	1.74	.18	.224
Shrub‐nesting bird abundance	−	**−0.247**	**13.53**	**.63**	**.006**		−1.854	0.91	.10	.367
Forest ground‐nesting bird abundance		0.028	0.62	.07	.453		−0.618	0.87	.09	.377
Tree‐nesting bird abundance	−	**−0.174**	**5.52**	**.41**	**.047**		−1.000	0.32	.04	.585
Cavity‐nesting bird abundance		0.134	2.00	.20	.194		−2.663	2.38	.23	.162
Foliage‐gleaning bird abundance		−0.259	4.62	.37	.064		−1.236	0.19	.02	.669
Ground‐foraging bird abundance		0.093	0.91	.10	.367		−3.079	3.78	.32	.088

	Shrub cover – 17%–82%					Ground cover diversity – 0.80–1.75				
		β	*F*_1,26_	*r* ^2^	*p*		β	*F*_1,26_	*r* ^2^	*p*
Total bird abundance		0.019	0.01	.00	.894		14.178	0.26	.03	.621
Bird species richness		−0.085	2.56	.24	.148		16.083	2.07	.20	.188
Chao richness estimator		−0.105	0.75	.08	.410		38.336	3.00	.27	.121
Shannon diversity	−	**0.005**	**8.05**	**.50**	**.022**		0.649	2.28	.22	.170
Shannon evenness	−	**‐5E‐04**	**15.85**	**.66**	**.004**		0.018	0.14	.02	.713
Shrub‐nesting bird abundance		0.105	2.05	.20	.190		−1.657	0.01	.00	.925
Forest ground‐nesting bird abundance		−0.005	0.03	.04	.868		−6.808	1.68	.17	.231
Tree‐nesting bird abundance		−0.033	0.22	.03	.655		1.664	0.01	.00	.915
Cavity‐nesting bird abundance		−0.048	0.39	.05	.549		19.727	1.78	.18	.219
Foliage‐gleaning bird abundance		0.101	0.88	.10	.380		−21.440	0.93	.10	.362
Ground‐foraging bird abundance		−0.023	0.09	.01	.776	+	**30.181**	**6.46**	**.45**	**.035**

**Figure 4:**
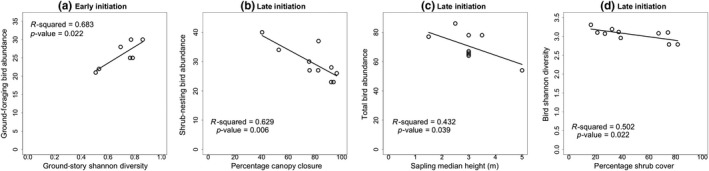
Regressions depict strong relationships between regenerating vegetation and bird community variables in early and late initiation. Figures depict the following relationships in early initiation: (a) ground‐foraging bird abundance against ground‐story Shannon diversity. Figures depict the following relationships in late initiation: (b) shrub‐nesting bird abundance against percent canopy cover, (c) total bird abundance against sapling median height, and (d) bird Shannon diversity against total shrub cover

Across all stand phases, sapling height negatively correlates with total bird abundance, richness, Shannon diversity, and—very strongly—shrub‐nesting and foliage‐gleaning bird abundances (Table 1). Within late initiation (LI), sapling height negatively correlates with total bird abundance (Table 1; Figure 4).

Across all stand phases, ground‐story plant Shannon diversity positively correlates with total bird abundance, bird species richness, Chao richness estimator, bird Shannon diversity, shrub‐nesting bird abundance, and foliage‐gleaning bird abundance and negatively correlates with forest ground‐nesting bird abundance (Table 1). Within early initiation (EI) and late initiation (LI), ground‐foraging bird abundance positively correlates with ground‐story Shannon diversity (*r* ^2^ = .683, *p *< .025; Table 1; Figure 4).

Across all stand phases, total shrub cover negatively correlates with bird Shannon evenness (Table 1). It bears hump‐shaped modal relationships with total bird abundance, bird species richness, bird Shannon diversity, and shrub‐nesting bird abundance, increasing then decreasing (Table 1). Maximum bird variables values occur between 30 and 50 percent shrub cover. Within late initiation (LI), total shrub cover negatively correlates with bird Shannon diversity and Shannon evenness (Table 1).

Across all stand phases, ground‐story Shannon diversity positively correlates with the abundance of the shrub‐nesting species common yellowthroat (*r* ^2^ = .22, *p* < .02), eastern towhee (*r* ^2^ = .15, *p* < .05), and gray catbird (*r* ^2^ = .39, *p* < .0005). The gray catbird also positively correlates with total shrub cover (*r* ^2^ = .26, *p* < .01). The towhee negatively correlates with sapling median height (*r* ^2^ = .14, *p* < .05), and both the towhee and the common yellowthroat negatively correlate with percent canopy cover (*r* ^2^ = .32, *p* < .002; *r* ^2^ = .34, *p* < .002, respectively). In contrast, the veery (*Catharus fuscescens*) positively correlates with canopy cover (*r* ^2^ = .16, *p* < .04).

### 3.2 Legacy tree effect

When subjected to ANOVA or Kruskal–Wallis tests, three of the five legacy stand variables did not change significantly with stand phase, supporting our assumption that they reflect stand characteristics that do not change with forest succession (Appendix S3). We attribute the changes of the two that varied with stand phase, legacy stem density (*F* = 4.26, *p* < .026) and total tree species richness (*F* = 6.86, *p* < .032), to, respectively, some saplings entering the legacy size class and mortality with stem competition.

**Figure 5:**
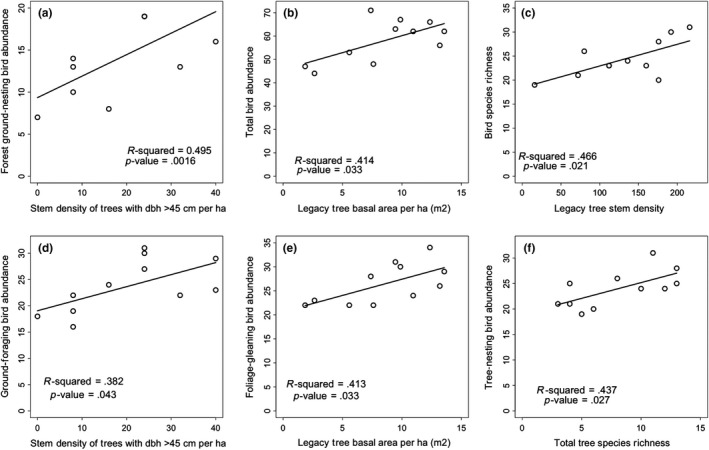
Regressions depict varied strong relationships between legacy stand vegetation and bird community variables in early stem exclusion. Figures depict the following relationships: (a) forest ground‐nesting bird abundance against stem density of trees with DBH >45 cm per ha, (b) total bird abundance against legacy tree basal area per ha, (c) bird species richness against legacy tree stem density per ha, (d) ground‐foraging bird abundance against stem density of trees with DBH >45 cm per ha, (e) foliage‐gleaning bird abundance against legacy tree basal area per ha, and (f) tree‐nesting bird abundance against tree species richness

Across the three phases of stand development, the stem density of trees with DBH >45 cm positively correlates with forest ground‐nesting bird abundance (Table 2). Within early stem exclusion (ESE), the stem density of trees with DBH >45 cm positively correlates with forest ground‐nesting bird abundance and ground‐foraging bird abundance (Table 2, Figure 5).

**Table 2:** Effects of legacy stand structure across stand phases and within early stem exclusion**.** Significant correlations in bold. Positive or rising quadratic correlations are denoted by plus signs and negative correlations by minus signs. Where the model is quadratic, an “*I*” value is present. For these models, the degrees of freedom are 2 and 25

Stand phase—All	# trees of dbh >45 cm 0–58 per hectare					Legacy tree basal area 0.6–16.5 m^2^ per hectare					Legacy stem density 8–225 per hectare					Tree species richness 1–13					Canopy structure diversity 0–1.28				
		β	*F*_1,26_	*r* ^2^	*p*		β	*F*_1,26_	*r* ^2^	*p*		β	*F*_1,26_	*r* ^2^	*p*		β	*F*_1,26_	*r* ^2^	*p*		β	*F*_1,26_	*r* ^2^	*p*
Total bird abundance		−0.63	0.21	.01	.65		−0.50	0.86	.03	.36		−0.48	2.77	.10	.11	**−**	**−1.43**	**5.94**	**.19**	**.02**		4.37	0.68	.03	.42
Bird species richness		−0.31	0.40	.02	.53		−0.04	0.04	.00	.85		0.04	0.14	.01	.71		−0.05	0.06	.00	.82	**+**	**4.32**	**6.55**	**.20**	**.02**
Chao richness		−0.52	0.36	.01	.55		0.04	0.01	.00	.91		0.18	0.97	.04	.33		−0.05	0.01	.00	.91		6.21	3.91	.13	.06
Shannon diversity		−0.01	0.43	.02	.52		0.00	0.04	.00	.84		0.00	0.31	.01	.58		0.00	0.16	.01	.70	**+**	**0.17**	**6.82**	**.21**	**.01**
Shannon evenness		0.00	0.19	.01	.67		0.00	0.10	.00	.75		0.00	0.23	.01	.63	**+**	**0.00**	**4.40**	**.14**	**.05**		0.01	0.62	.02	.44
Shrub‐nesting bird abundance		−0.09	0.36	.01	.56		−0.79	2.87	.10	.10	**−**	**−0.08**	**6.99**	**.21**	**.01**	**‐**	**−1.66**	**11.43**	**.31**	**.00**		−36.05	2.94	.19	.07
Forest ground‐nesting bird abundance	**+**	**0.16**	**8.93**	**.26**	**.01**	**+**	**0.51**	**9.88**	**.28**	**.00**		0.02	2.49	.09	.13		0.16	0.50	.02	.49		1.35	0.52	.02	.48
Tree‐nesting bird abundance		−0.50	1.07	.04	.31		−0.15	0.61	.02	.44		−0.05	0.23	.01	.63		0.04	0.02	.00	.88		0.99	0.27	.01	.61
Foliage‐gleaning bird abundance		−0.60	0.37	.01	.55		−0.65	3.05	.11	.09	**−**	**−0.60**	**10.59**	**.29**	**.00**	**−**	**−1.36**	**12.54**	**.33**	**.00**		−3.83	1.03	.04	.32
Ground‐foraging bird abundance		0.19	0.11	.00	.74		0.17	0.62	.02	.44		0.10	0.72	.03	.40		−0.01	0.00	.00	.97	**+**	**−1.42**	**6.08**	**.33**	**.01**

Stand phase—early Stem exclusion	# trees of dbh >45 cm 0–58 per hectare					Legacy tree basal area 0.6–16.5 m^2^ per hectare						Legacy stem density 8–225 per hectare					Tree species richness 1–13				Canopy structure diversity 0–1.28				
		β	*F*_1,9_	*r* ^2^	*p*		β	*F*_1,9_	*r* ^2^	*p*		β	*F*_1,9_	*r* ^2^	*p*		β	*F*_1,9_	*r* ^2^	*p*		β	*F*_1,9_	*r* ^2^	*p*
Total bird abundance		2.70	3.09	.26	.11	**+**	**1.45**	**6.35**	**.41**	**.03**		0.48	1.67	.16	.23		−0.22	0.08	.01	.79		10.35	1.98	.18	.19
Bird species richness		0.69	0.86	.09	.38		0.52	3.36	.27	.10	**+**	**0.36**	**7.84**	**.47**	**.02**		0.24	0.52	.05	.49		3.91	1.39	.13	.27
Chao richness estimator		1.37	0.74	.08	.41		0.73	1.22	.12	.30		0.49	2.09	.19	.18		−0.20	0.07	.01	.79		5.50	0.56	.06	.47
Shannon diversity		0.02	0.42	.04	.53		0.02	2.05	.19	.19	**+**	**0.01**	**6.21**	**.41**	**.03**		0.02	1.78	.17	.21		0.17	1.71	.16	.22
Shannon evenness		0.00	0.72	.07	.42		0.00	0.60	.06	.46		0.00	0.00	.00	.98		0.00	3.70	.29	.09		0.01	0.27	.03	.62
Shrub‐nesting bird abundance		0.17	2.33	.21	.16		0.34	0.75	.08	.41		0.00	0.00	.00	.95		−0.58	2.34	.21	.16		1.09	0.06	.01	.81
Forest ground‐nesting bird abundance	**+**	**0.26**	**8.81**	**.49**	**.02**		0.61	2.93	.25	.12		−0.01	0.17	.02	.69		−0.56	2.15	.19	.18		2.21	0.26	.03	.63
Tree‐nesting bird abundance		−0.70	1.08	.11	.33		0.15	0.27	.03	.62		0.25	3.44	.28	.10	**+**	**0.62**	**6.99**	**.44**	**.03**		4.71	2.76	.23	.13
Cavity‐nesting bird abundance		0.19	0.09	.01	.77		0.35	2.10	.19	.18		0.26	4.91	.35	.05		0.17	0.37	.04	.56		1.88	0.44	.05	.52
Foliage‐gleaning bird abundance		0.95	1.54	.15	.25	**+**	**0.68**	**6.34**	**.41**	**.03**		0.25	2.29	.20	.16		0.60	4.12	.31	.07		−0.16	0.00	.00	.97
Ground‐foraging bird abundance	**+**	**1.83**	**5.57**	**.38**	**.04**		0.55	2.16	.19	.18		0.01	0.00	.00	.98		0.63	2.90	.24	.12		5.14	1.51	.14	.25

Across the three stand phases, legacy tree basal area positively correlates with forest ground‐nesting bird abundance (Table 2). Within early stem exclusion (ESE), it positively correlates with total bird abundance and foliage‐gleaning bird abundance (Table 2, Figure 5).

Across the three stand phases, legacy tree stem density positively correlates with forest ground‐nesting bird abundance and negatively correlates with shrub‐nesting and foliage‐gleaning bird abundances (Table 2). Within early stem exclusion, legacy stem density positively correlates with bird species richness and Shannon diversity (Table 2, Figure 5).

Across the three stand phases, total tree species richness positively correlates with bird Shannon evenness and negatively correlates with total bird abundance, shrub‐nesting bird abundance, and foliage‐gleaning bird abundance (Table 2). Within early stem exclusion (ESE), total tree species richness positively correlates to the abundance of forest ground‐nesting birds (Table 2, Figure 5).

Across stand phases, canopy structure Shannon diversity positively correlates with bird species richness, bird Shannon diversity, and ground‐foraging bird abundance (Table 2, Figure 4). Within late initiation, it positively correlates with bird species richness. Canopy structure diversity does not correlate significantly with any other vegetation variables over both linear and binomial regression.

The common yellowthroat and eastern towhee negatively correlate with legacy stem density (*r* ^2^ = .23, *p* < .01; *r* ^2^ = .16, *p* < .04) and total tree species richness (*r* ^2^ = .32, *p* < .002; *r* ^2^ = .37, *p* < .001). The veery positively correlates with the stem density of trees with DBH >45 cm and legacy tree basal area (*r* ^2^ = .17, *p* < .03; *r* ^2^ = .16, *p* < .04). Notably, during early initiation (EI), the wood thrush (*Hylocichla mustelina*) positively correlates with the stem density of trees with DBH >45 cm (*r* ^2^ = .74, *p* < .014). During early stem exclusion (ESE), the red‐eyed vireo (*Vireo olivaceus*) positively correlates with canopy structure diversity (*r* ^2^ = .51, *p* < .014).

Hierarchical partitioning supported the relative significance and independent effects of all vegetation variables in relation to response variables.

## 4 DISCUSSION

Results from this study suggest that diverse groundcover, dense shrub cover, and legacy stand canopy structure diversity result in a richer and more abundant bird community. The strengths of these separate influences change with forest succession.

### 4.1 Regenerating stand structure

Our results show that changes in regenerating vegetation bear primarily negative relationships with bird community variables through all stand phases and particularly during late initiation (LI). Notably, ground‐story Shannon diversity is the most consistently powerful correlate of bird community variables.

The evidence for the strength of temporal effects postharvest on the bird community is substantial. Multiple studies have examined the responses of bird functional guilds to harvest and time since harvest (Duguid et al., 2016; Newell & Rodewald, 2011; Preston & Harestad, 2007; Tozer, Burke, Nol, & Elliott, 2010). Previous studies of the effects of variable retention harvests have suggested that the successional trajectory of the forest drives bird community composition and that bird species richness and abundance decline in stem exclusion (Duguid et al., 2016; Grodsky, Moorman, Fritts, Castleberry, & Wigley, 2016; Winkler, 2005). As reported in other studies, our results show that bird abundance within the shrub‐nesting functional guild decreases swiftly as the stand regenerates postharvest. This guild contains species declining in northeastern North America and comprises nearly half of total bird abundance that we recorded in early stand initiation (Holmes & Sherry, 2001; Holmes et al., 1986). While other studies correlated bird variables with time, this study correlates them with changing stand structure.

Our results show that regenerating vegetation variables—particularly percent canopy cover—capture aspects of stand development that bear specific relationships with nesting and foraging functional guilds. Consistent with other studies, our results demonstrate that while some birds decline with forest succession, others increase (Begehold, Rzanny, & Flade, 2014; Duguid et al., 2016). Separately, we note that high shrub‐nesting bird abundance and the presence of forest ground‐nesting birds during early and late initiation stages of stand development support literature that asserts that newly open areas create habitat for both juvenile early‐successional‐associated and forest‐associated species (Chandler, King, & Chandler, 2012; Schlossberg, 2009). The marked strength of the correlations between percent canopy cover and bird community variables supports the theory that canopy closure plays a significant role in driving changes in bird community (King & DeGraaf, 2000). For example, our study showed that shrub‐nesting, foliage‐gleaning, and total bird abundance initially increase slightly and then progressively decrease in relation to increasing canopy cover. Studies have attributed similar shrub‐nesting bird response patterns to increases in insects, theorizing that insects increase in a newly opened stand as herb and young seedling cover expands and temperatures are higher, but then subsequently with canopy closure and dominance of more woody plants, food plants are less palatable and insect populations decline (Table 1, Figure 3; Hilmers et al., 2018).

The fact that ground‐story diversity is the most consistent correlate among the regenerating vegetation variables suggests that forage and nesting habitat diversity positively drives bird community composition and size. At the Yale‐Myers Forest, a mixed groundcover includes shrubs that accumulate and shade leaf litter and support rich insect communities, providing food, nesting cover, and nesting material (Duguid et al., 2013). Studies conflict on the response of ground‐story plant species diversity and abundance to harvest. Some research suggests that richness and abundance of ground‐story plants increases with amount of retained structure; and that functional composition of the ground‐story changes from fruiting annual herbs and forbs to immature perennial woody plants with time since harvest (Duguid et al., 2013; Lilles, Dhar, Coates, & Haeussler, 2018; Macdonald & Fenniak, 2007; Scheller & Mladenoff, 2002; Zenner, Kabrick, Jensen, Peck, & Grabner, 2006)*.* In our results, a positive relationship between foliage‐gleaning bird abundance and ground‐story diversity and very strong relationships between ground‐foraging bird abundance and ground‐story diversity during early stand initiation (*r* ^2^ = .74, *p *< .02) and late stand initiation suggest that ground‐story diversity may be particularly important for forage.

Our results are supported by other studies that demonstrate the importance of the ground‐story suggesting that high diversity and moderate amounts of shrub cover would result in the greatest bird species richness and abundance. For example, Schlossberg, King, Chandler, and Mazzei (2010) concluded that bird‐preferred understory habitat could be separated into areas of tall shrub cover or low shrubs and forbs. Other studies show that some shrub‐nesting birds prefer sites with high woody stem density as predation defense (Rockwell & Stephens, 2017; Schill & Yahner, 2009; Stauffer & Best, 1986). As the ground‐foraging birds in our survey are almost entirely insect—consumers or omnivores, we suggest that ground‐foraging bird abundance may be due to the abundance and diversity of prey—and that a relationship between canopy and ground‐story arthropods, and their changes in abundance and composition over stand development bear further investigation.

Relationships within late stand initiation between regenerating vegetation and bird community variables show that correlations become particularly strong as the stand shifts from initiation to stem exclusion (see Figure 4). They provide evidence to support the importance of developmental phase to the drivers of bird community composition (Hutto, 1995; Welsh & Healy, 1993).

### 4.2 Legacy stand structure

Across all three stand phases and particularly during early stem exclusion, legacy vegetation variables bear primarily positive relationships with bird community variables, particularly forest ground‐nesting bird abundance. In exception, shrub‐nesting birds have negative relationships with legacy stem density and tree species richness.

Our results suggest that trees with DBH >45 cm may increase bird abundance and diversity via nesting habitat and forage (Table 2). Literature suggests the large trees act as keystone habitat features (DeMars, Rosenberg, & Fontaine, 2010). The rough, corrugated bark, and multiple microhabitats, like small places of rot, of larger trees host insects, providing rich and abundant forage for birds of multiple guilds (Großmann, Schultze, Bauhus, & Pyttel, 2018; Kozák et al., 2018; Larrieu & Cabanettes, 2012). Studies have tied specifically bark‐foraging bird species to large‐diameter trees (Pennington & Blair, 2011; Whelan & Maina, 2005). Our results are novel in that they tie ground‐foraging and ground‐nesting birds to trees with DBH >45 cm. The latter suggests that large trees may play an unexpected role in supporting this guild, perhaps by providing pockets of forest ground excluded from the dense stem competition of early stem exclusion. A correlation between large‐diameter tree stem density with abundance of cavity‐nesting bird abundance (e.g., woodpeckers), found in multiple forest types in America and Europe and attributed to large‐diameter trees providing potential nesting sites, is absent from these data (Anderson & Shugart, 1974; Gutzat & Dormann, 2018; Poulsen, 2002). We may find an explanation for the absence of this relationship in a study that identified smaller diameter trees as preferable for bark‐foraging cavity‐nesters, so that they may avoid threat of predation with a greater field of vision (Whelan & Maina, 2005).

Canopy structure diversity plays a significant role in bird community composition within our study. Importantly, canopy structure diversity neither changes with stand phase nor does it bear significant relationships with other vegetation variables. While Kane et al. (2011) associated stand structure with phases in stand development by interpreting combinations of stand structure and canopy cover, Donato, Campbell, and Franklin (2011) proposed that forest succession can include spatial complexity from initiation and our data support the argument that canopy structure diversity can be independent of stand phase. The positive correlation between canopy structure diversity and bird species richness is consistent with the results of a study that used lidar data to relate bird species richness with vertical canopy distribution (Goetz, Steinberg, Dubayah, & Blair, 2007). We suggest this correlation and that of bird Shannon diversity with canopy structure diversity may be due to changes in prey—the arthropods whose abundance, richness, and composition vary with canopy structure complexity and location within the canopy (Table 2, Figure 4) (Aikens & Buddle, 2012; Halaj, Ross, & Moldenke, 2000; Ulyshen, 2011). We note that the stands with greatest canopy structure diversity have between four and five canopy layers with at least one canopy layer comprising more than a third as many stems as those that comprise the layer in highest canopy.

Most interestingly, our results show that, during early stem exclusion—the most depauperate phase of early stand development in regard to stand structure—leaving legacy stand structure correlates with bird community variables (Table 2) (Hilmers et al., 2018). Notably, the relationship between foliage‐gleaning bird abundance and legacy tree basal area reverses over stand succession, switching from a strong negative correlation (*r* ^2^ = .59, *p* < .04) to a positive one. As other studies have observed, forage diversity may account for the strong correlation during early stem exclusion between diversity in tree structure and tree‐nesting bird abundance, but more research is needed on this relationship (Gil‐tena, Saura, & Brotons, 2007; Poulsen, 2002). In sum, these relationships suggest that legacy stand structure is particularly important during early stem exclusion and supports the stand phase model as an identification of successional periods when processes vary in explanatory power.

## 5 CONCLUSION

Our results support temperate mixed broadleaf management to promote ground‐story diversity and retain varied legacy stand structure. They agree with prior research in demonstrating that the ephemerality of early seral habitat requires landscape‐level management—harvests timed in intervals of 7–10 years across a forest—but we demonstrate the importance of legacy stand structure during early stem exclusion and its relationships with a variety of bird functional guilds. These findings are new and elaborate upon other studies by Holmes et al. (1986), Holmes and Sherry (2001), Morris et al. (2013), and Fedrowitz et al. (2014), by clearly showing what vegetative structures and phases of stand dynamics either promote or negatively impact breeding bird diversity and abundance. Based on our results, we recommend that irregular shelterwoods in temperate oak‐mixed broadleaf forests like those of northeastern Connecticut retain at least 20 trees of DBH >45 cm per ha, at least 7 m^2^ of legacy tree basal area per ha, and trees of varied canopy layers with at least one layer containing a third the number of stems in the canopy. Our results showing the benefits of retaining varied legacy tree species and structures to increase bird abundance and diversity in shelterwoods complement other management objectives that include better water conservation, climate resilience, and timber yield (Ashton & Kelty, 2018; Fedrowitz et al., 2014). We contribute to research that demonstrates the effects of increasing heterogeneity of stand structure on bird diversity (Bae et al., 2018; Dobson, Sorte, Manne, & Hawkins, 2015; Hewson, Austin, Gough, & Fuller, 2011; James & Wamer, 1982; MacArthur & MacArthur, 1961; Robinson & Holmes, 1982; Verschuyl, Hansen, McWethy, Sallabanks, & Hutto, 2008).

Forestry, particularly that of irregular shelterwoods, requires managing multiple timelines across a landscape. By retaining diverse legacy stand structure, foresters and land managers can ensure that a stand hosts diverse and abundant birds during a depauperate phase of development.

## AUTHOR CONTRIBUTION

MA conceived the framework, JH collected the bird data and ran the analyses, and MD contributed to the sampling protocol and provided substantial advice in analysis and interpretation. JH wrote the bulk of the manuscript with substantial advice and edits from MA and MD.

## Data Availability

Data from this study are available and can be accessed at the public data repository Dryad. DOI accession number—https://doi.org/10.5061/dryad.n8pk0p2rb.

## References

[ece35967-bib-0001] Aikens, K. , & Buddle, C. (2012). Small‐scale heterogeneity in temperate forest canopy arthropods: Stratification of spider and beetle assemblages. The Canadian Entomologist, 144(04), 526–537. 10.4039/tce.2012.51

[ece35967-bib-0003] Anderson, S. H. , & Shugart, H. H. (1974). Habitat selection of breeding birds in an east Tennessee deciduous forest. Ecology, 55(4), 828–837. 10.2307/1934418

[ece35967-bib-0004] Ashton, M. S. , Duguid, M. C. , Barrett, A. L. , & Covey, K. (2015). Yale School Forests, New England, United States of America In Siry J. P., Bettinger P., Merry K., Grebner D. L., Boston K., & Cieszewski C. (Eds.), Forest plans of North America (pp. 253–264). New York, NY: Academic Press.

[ece35967-bib-0005] Ashton, M. S. , & Kelty, M. J. (2018). The practice of silviculture: Applied forest ecology (10th ed.). New York, NY: John Wiley and Sons.

[ece35967-bib-0006] Askins, R. A. (1998). Restoring forest disturbances to sustain populations of shrubland birds. Ecological Restoration, 16(2), 166–173. 10.3368/er.16.2.166

[ece35967-bib-0007] Askins, R. A. , Zuckerberg, B. , & Novak, L. (2007). Do the size and landscape context of forest openings influence the abundance and breeding success of shrubland songbirds in southern New England? Forest Ecology and Management, 250(3), 137–147. 10.1016/j.foreco.2007.05.009

[ece35967-bib-0008] Bae, S. , Müller, J. , Lee, D. , Vierling, K. T. , Vogeler, J. C. , Vierling, L. A. , … Thorn, S. (2018). Taxonomic, functional, and phylogenetic diversity of bird assemblages are oppositely associated to productivity and heterogeneity in temperate forests. Remote Sensing of Environment, 215, 145–156. 10.1016/j.rse.2018.05.031

[ece35967-bib-0009] Begehold, H. , Rzanny, M. , & Flade, M. (2014). Forest development phases as an integrating tool to describe habitat preferences of breeding birds in lowland beech forests. Journal of Ornithology, 156(1), 19–29. 10.1007/s10336-014-1095-z

[ece35967-bib-0011] Bishop, J. , & Myers, W. (2005). Associations between avian functional guild response and regional landscape properties for conservation planning. Ecological Indicators, 5(1), 33–48. 10.1016/j.ecolind.2004.10.001

[ece35967-bib-0012] Block, W. M. , & Brennan, L. A. (1993). The habitat concept in ornithology Current ornithology (pp. 35–91). Boston, MA: Springer.

[ece35967-bib-0014] Brawn, J. D. , Robinson, S. K. , & Thompson, F. R. (2001). The ole of disturbance in the ecology and conservation of birds. Annual Review of Ecology and Systematics, 32(1), 251–276. 10.1146/annurev.ecolsys.32.081501.114031

[ece35967-bib-0015] Brunet, J. , Valtinat, K. , Mayr, M. , Felton, A. , Lindbladh, M. , & Bruun, H. (2011). Understory succession in post‐agricultural oak forests: Habitat fragmentation affects forest specialists and generalists differently. Forest Ecology and Management, 262(9), 1863–1871. 10.1016/j.foreco.2011.08.007

[ece35967-bib-0016] Chandler, C. , King, D. , & Chandler, R. (2012). Do mature forest birds prefer early‐successional habitat during the post‐fledging period? Forest Ecology and Management, 264, 1–9. 10.1016/j.foreco.2011.09.018

[ece35967-bib-0017] Cody, M. L. (Ed.) (1985). Habitat selection in birds (p. 558). New York, NY: Academic Press.

[ece35967-bib-0018] DeGraaf, R. M. , & Yamasaki, M. (2003). Options for managing early‐successional forest and shrubland bird habitats in the northeastern United States. Forest Ecology and Management, 185(1–2), 179–191. 10.1016/s0378-1127(03)00254-8

[ece35967-bib-0019] DeMars, C. , Rosenberg, D. , & Fontaine, J. (2010). Multi‐scale factors affecting bird use of isolated remnant oak trees in agro‐ecosystems. Biological Conservation, 143(6), 1485–1492. 10.1016/j.biocon.2010.03.029

[ece35967-bib-0021] Dobson, L. , Sorte, F. , Manne, L. , & Hawkins, B. (2015). The diversity and abundance of North American bird assemblages fail to track changing productivity. Ecology, 96(4), 1105–1114. 10.1890/14-0057.1 26230030

[ece35967-bib-0022] Donato, D. , Campbell, J. , & Franklin, J. (2011). Multiple successional pathways and precocity in forest development: Can some forests be born complex? Journal of Vegetation Science, 23(3), 576–584. 10.1111/j.1654-1103.2011.01362.x

[ece35967-bib-0023] Donner, D. M. , Ribic, C. A. , & Probst, J. R. (2010). Patch dynamics and the timing of colonization–abandonment events by male Kirtland's Warblers in an early succession habitat. Biological Conservation, 143(5), 1159–1167. 10.1016/j.biocon.2010.02.023

[ece35967-bib-0024] Donoso, P. J. , & Nyland, R. D. (2006). Interference to hardwood regeneration in northeastern North America: The effects of raspberries (*Rubus* sp.) following clearcutting and shelterwood methods. Northern Journal of Applied Forestry, 23, 288–296. 10.1093/njaf/23.4.288

[ece35967-bib-0026] Duguid, M. C. , Frey, B. R. , Ellum, D. S. , Kelty, M. J. , & Ashton, M. S. (2013). The influence of ground disturbance and gap position on understory plant diversity in upland forests of southern New England. Forest Ecology and Management, 303, 148–159. 10.1016/j.foreco.2013.04.018

[ece35967-bib-0027] Duguid, M. C. , Morrell, E. H. , Goodale, E. , & Ashton, M. S. (2016). Changes in breeding bird abundance and species composition over a 20 year chronosequence following shelterwood harvests in oak‐hardwood forests. Forest Ecology and Management, 376, 221–230. 10.1016/j.foreco.2016.06.010

[ece35967-bib-0028] Fedrowitz, K. , Koricheva, J. , Baker, S. , Lindenmayer, D. , Palik, B. , Rosenvald, R. , … Messier, C. (2014). Can retention forestry help conserve biodiversity? A meta‐analysis. Journal of Applied Ecology, 51(6), 1669–1679. 10.1111/1365-2664.12289 25552747 PMC4277688

[ece35967-bib-0029] Foster, D. R. , Motzkin, G. , & Slater, B. (1998). Land‐use history as long‐term broad‐scale disturbance: Regional forest dynamics in central New England. Ecosystems, 1(1), 96–119. 10.1007/s100219900008

[ece35967-bib-0030] Fredericksen, T. S. , Ross, B. D. , Hoffman, W. , Morrison, M. L. , Beyea, J. , Johnson, B. N. , … Ross, E. (1999). Short‐term understory plant community responses to timber‐harvesting intensity on non‐industrial private forestlands in Pennsylvania. Forest Ecology and Management, 116(1–3), 129–139. 10.1016/s0378-1127(98)00452-6

[ece35967-bib-0031] Frey, B. , Ashton, M. S. , McKenna, J. , Ellum, D. S. , & Finkral, A. J. (2007). Topographic and temporal patterns in tree seedling establishment, growth, and survival among masting species of southern New England mixed‐deciduous forests. Forest Ecology and Management, 245(1–3), 54–63. 10.1016/j.foreco.2007.03.069

[ece35967-bib-0032] Gil‐Tena, A. , Saura, S. , & Brotons, L. (2007). Effects of forest composition and structure on bird species richness in a Mediterranean context: Implications for forest ecosystem management. Forest Ecology and Management, 242(2–3), 470–476. 10.1016/j.foreco.2007.01.080

[ece35967-bib-0033] Goetz, S. , Steinberg, D. , Dubayah, R. , & Blair, B. (2007). Laser remote sensing of canopy habitat heterogeneity as a predictor of bird species richness in an eastern temperate forest, USA. Remote Sensing of Environment, 108(3), 254–263. 10.1016/j.rse.2006.11.016

[ece35967-bib-0034] Goodale, E. , Lalbhai, P. , Goodale, U. M. , & Ashton, P. M. S. (2009). The relationship between shelterwood cuts and crown thinnings and the abundance and distribution of birds in a southern New England forest. Forest Ecology and Management, 258(3), 314–322. 10.1016/j.foreco.2009.04.020

[ece35967-bib-0035] Grodsky, S. , Moorman, C. , Fritts, S. , Castleberry, S. , & Wigley, T. (2016). Breeding, early‐successional bird response to forest harvests for bioenergy. PLoS ONE, 11(10), e0165070 10.1371/journal.pone.0165070 27780221 PMC5079583

[ece35967-bib-0036] Großmann, J. , Schultze, J. , Bauhus, J. , & Pyttel, P. (2018). Predictors of Microhabitat Frequency and Diversity in Mixed Mountain Forests in South‐Western Germany. Forests, 9(3), 104 10.3390/f9030104

[ece35967-bib-0037] Gustafsson, L. , Baker, S. C. , Bauhus, J. , Beese, W. J. , Brodie, A. , Kouki, A. B. J. , … Franklin, J. F. (2012). Retention forestry to maintain multifunctional forests: A world perspective. BioScience, 62(7), 633–645. 10.1525/bio.2012.62.7.6

[ece35967-bib-0038] Gutzat, F. , & Dormann, C. (2018). Decaying trees improve nesting opportunities for cavity‐nesting birds in temperate and boreal forests: A meta‐analysis and implications for retention forestry. Ecology and Evolution, 8(16), 8616–8626. 10.1002/ece3.4245 30250728 PMC6144968

[ece35967-bib-0039] Halaj, J. , Ross, D. , & Moldenke, A. (2000). Importance of habitat structure to the arthropod food‐web in Douglas‐fir canopies. Oikos, 90(1), 139–152. 10.1034/j.1600-0706.2000.900114.x

[ece35967-bib-0040] Hewson, C. , Austin, G. , Gough, S. , & Fuller, R. (2011). Species‐specific responses of woodland birds to stand‐level habitat characteristics: The dual importance of forest structure and floristics. Forest Ecology and Management, 261(7), 1224–1240. 10.1016/j.foreco.2011.01.001

[ece35967-bib-0041] Hilmers, T. , Friess, N. , Bässler, C. , Heurich, M. , Brandl, R. , Pretzsch, H. , … Muller, J. (2018). Biodiversity along temperate forest succession. Journal of Applied Ecology, 55(6), 2756–2766. 10.1111/1365-2664.13238

[ece35967-bib-0042] Holmes, R. T. , Bonney, R. , & Pacala, S. (1979). Guild Structure of the Hubbard Brook bird community: A multivariate approach. Ecology, 60(3), 512–520. 10.2307/1936071

[ece35967-bib-0043] Holmes, R. W. , & Sherry, T. W. (2001). Thirty‐year bird population trends in an unfragmented temperate deciduous forest: Importance of habitat change. The Auk, 118(3), 589–609. 10.2307/4089923

[ece35967-bib-0044] Holmes, R. W. , Sherry, T. W. , & Sturges, F. W. (1986). Bird community dynamics in a temperate deciduous forest: Long‐term trends at Hubbard Brook. Ecological Monographs, 56(3), 201–220. 10.2307/2937074

[ece35967-bib-0045] Hutto, R. L. (1995). Composition of bird communities following stand‐replacement fires in northern Rocky Mountain (USA) conifer forests. Conservation Biology, 9(5), 1041–1058. 10.1046/j.1523-1739.1995.9051033.x-i1 34261259

[ece35967-bib-0046] James, F. C. , & Wamer, N. O. (1982). Relationships between temperate forest bird communities and vegetation structure. Ecology, 63(1), 159–171. 10.2307/1937041

[ece35967-bib-0047] Johnston, D. W. , & Odum, E. P. (1956). Breeding bird populations in relation to plant succession on the Piedmont of Georgia. Ecology, 37(1), 50–62. 10.2307/1929668

[ece35967-bib-0048] Kane, V. , Gersonde, R. , Lutz, J. , McGaughey, R. , Bakker, J. , & Franklin, J. (2011). Patch dynamics and the development of structural and spatial heterogeneity in Pacific Northwest forests. Canadian Journal of Forest Research, 41(12), 2276–2291. 10.1139/x11-128

[ece35967-bib-0049] Keller, J. K. , Richmond, M. E. , & Smith, C. R. (2003). An explanation of patterns of breeding bird species richness and density following clearcutting in northeastern USA forests. Forest Ecology and Management, 174(1–3), 541–564. 10.1016/s0378-1127(02)00074-9

[ece35967-bib-0051] King, D. I. , & DeGraaf, R. M. (2000). Bird species diversity and nesting success in mature, clearcut and shelterwood forest in northern New Hampshire, USA. Forest Ecology and Management, 129(1–3), 227–235. 10.1016/s0378-1127(99)00167-x

[ece35967-bib-0053] King, D. I. , & Schlossberg, S. (2014). Synthesis of the conservation value of the early‐successional stage in forests of eastern North America. Forest Ecology and Management, 324, 186–195. 10.1016/j.foreco.2013.12.001

[ece35967-bib-0054] Kozák, D. , Mikoláš, M. , Svitok, M. , Bače, R. , Paillet, Y. , Larrieu, L. , … Svoboda, M. (2018). Profile of tree‐related microhabitats in European primary beech‐dominated forests. Forest Ecology and Management, 429, 363–374. 10.1016/j.foreco.2018.07.021

[ece35967-bib-0055] Kuuluvainen, T. , Leinonen, K. , Nygren, M. , & Penttinen, A. (1996). Statistical opportunities for comparing stand structural heterogeneity in managed and primeval forests: An example from boreal spruce forest in southern Finland. Silva Fennica, 30(2–3), 315–328. 10.14214/sf.a9243

[ece35967-bib-0056] Labbe, M. A. , & King, D. I. (2014). The effect of local and landscape‐level characteristics on the abundance of forest birds in early‐successional habitats during the post‐fledging season in western Massachusetts. PLoS ONE, 9(8), e106398 10.1371/journal.pone.0106398 25170610 PMC4149558

[ece35967-bib-0057] Larrieu, L. , & Cabanettes, A. (2012). Species, live status, and diameter are important tree features for diversity and abundance of tree microhabitats in subnatural montane beech–fir forests1This article is one of a selection of papers from the International Symposium on Dynamics and Ecological Services of Deadwood in Forest Ecosystems. Canadian Journal of Forest Research, 42(8), 1433–1445. 10.1139/x2012-077

[ece35967-bib-0058] Lilles, E. , Dhar, A. , Coates, K. , & Haeussler, S. (2018). Retention level affects dynamics of understory plant community recovery in northern temperate hemlock‐cedar forests. Forest Ecology and Management, 421, 3–15. 10.1016/j.foreco.2017.12.033

[ece35967-bib-0059] Litvaitis, J. A. (1993). Response of early successional vertebrates to historic changes in land use. Conservation Biology, 7(4), 866–873. 10.1046/j.1523-1739.1993.740866.x

[ece35967-bib-0060] Mac Nally, R. (1996). Hierarchical partitioning as an interpretative tool in multivariate inference. Australian Journal of Ecology, 21, 224–228. 10.1111/j.1442-9993.1996.tb00602.x

[ece35967-bib-0061] Mac Nally, R. (2002). Multiple regression and inference in ecology and conservation biology: Further comments on identifying important predictor variables. Biodiversity and Conservation, 11(8), 1397–1401. 10.1023/A:1016250716679

[ece35967-bib-0062] MacArthur, R. H. , & MacArthur, J. W. (1961). On bird species diversity. Ecology, 42(3), 594–598. 10.2307/1932254

[ece35967-bib-0063] Macdonald, S. , & Fenniak, T. (2007). Understory plant communities of boreal mixedwood forests in western Canada: Natural patterns and response to variable‐retention harvesting. Forest Ecology and Management, 242(1), 34–48. 10.1016/j.foreco.2007.01.029

[ece35967-bib-0064] Man, R. , & Yang, H. (2015). Construction of neighbourhood diversity indices with stem mapping data. Canadian Journal of Forest Research, 45(8), 1137–1141. 10.1139/cjfr-2015-0108

[ece35967-bib-0065] Morris, D. L. , Porneluzi, P. A. , Haslerig, J. , Clawson, R. L. , & Faaborg, J. (2013). Results of 20 years of experimental forest management on breeding birds in Ozark forests of Missouri, USA. Forest Ecology and Management, 310, 747–760. 10.1016/j.foreco.2013.09.020

[ece35967-bib-0066] Newell, F. , & Rodewald, A. (2011). Management for oak regeneration: Short‐term effects on the bird community and suitability of shelterwood harvests for canopy songbirds. The Journal of Wildlife Management, 76(4), 683–693. 10.1002/jwmg.314

[ece35967-bib-0067] North American Bird Conservation Initiative US Committee (2014). The state of the birds 2014 report. Washington, DC: US Department of Interior.

[ece35967-bib-0068] Oksanen, J. , Guillaume Blanchet, F. , Friendly, M. , Kindt, R. , Legendre, P. , McGlinn, D. , … Wagner, H. (2018). vegan: Community Ecology Package. R package version 2.5‐3. Retrieved from https://CRAN.R-project.org/package=vegan

[ece35967-bib-0069] Oliver, C. D. , & Larson, B. C. (1990). Forest stand dynamics. New York, NY: McGraw‐Hill Inc.

[ece35967-bib-0070] Oliver, C. D. , Larson, B. C. , & Oliver, C. D. (1996). Forest stand dynamics (p. 520). New York: Wiley.

[ece35967-bib-0072] Pennington, D. , & Blair, R. (2011). Habitat selection of breeding riparian birds in an urban environment: Untangling the relative importance of biophysical elements and spatial scale. Diversity and Distributions, 17(3), 506–518. 10.1111/j.1472-4642.2011.00750.x

[ece35967-bib-0073] Perry, R. W. , & Thill, R. E. (2013). Long‐term responses of disturbance‐associated birds after different timber harvests. Forest Ecology and Management, 307, 274–283. 10.1016/j.foreco.2013.07.026

[ece35967-bib-0074] Poulsen, B. O. (2002). Avian richness and abundance in temperate Danish forests: Tree variables important to birds and their conservation. Biodiversity and Conservation, 11(9), 1551–1566. 10.1023/A:1016839518172

[ece35967-bib-0075] Preston, M. , & Harestad, A. (2007). Community and species responses by birds to group retention in a coastal temperate forest on Vancouver Island, British Columbia. Forest Ecology and Management, 243(1), 156–167. 10.1016/j.foreco.2007.03.002

[ece35967-bib-0076] Pretzsch, H. , & Schütze, G. (2008). Transgressive overyielding in mixed compared with pure stands of Norway spruce and European beech in Central Europe: Evidence on stand level and explanation on individual tree level. European Journal of Forest Research, 128(2), 183–204. 10.1007/s10342-008-0215-9

[ece35967-bib-0077] R Core Team (2018). R: A language and environment for statistical computing. Vienna, Austria: R Foundation for Statistical Computing Retrieved from https://www.R-project.org/

[ece35967-bib-0078] Raymond, P. , Bédard, S. , Roy, V. , Larouche, C. , & Tremblay, S. (2009). The irregular shelterwood system: Review, classification, and potential application to forests affected by partial disturbances. Journal of Forestry, 107(8), 405–413. 10.1093/jof/107.8.405

[ece35967-bib-0079] Robbins, C. J. , Sauer, J. R. , & Droege, S. (1997). Monitoring bird populations by point counts. United States Forest Service, General Technical Report PSW‐GTR‐149, Albany, California, U.S. Department of Agriculture, Forest Service, Pacific Southwest Research Station. 187 p.

[ece35967-bib-0080] Robinson, S. , & Holmes, R. (1982). Foraging behavior of forest birds: The relationships among search tactics, Diet, and Habitat Structure. Ecology, 63(6), 1918 10.2307/1940130

[ece35967-bib-0081] Rockwell, S. , & Stephens, J. (2017). Habitat selection of riparian birds at restoration sites along the Trinity River, California. Restoration Ecology, 26(4), 767–777. 10.1111/rec.12624

[ece35967-bib-0082] RStudio Team (2016). RStudio: Integrated development for R. Boston, MA: RStudio Inc Retrieved from http://www.rstudio.com/

[ece35967-bib-0083] Scheller, R. M. , & Mladenoff, D. J. (2002). Understory species patterns and diversity in old‐growth and managed northern hardwood forests. Ecological Applications, 12(5), 1329–1343. 10.1890/1051-0761(2002)012[1329:Uspadi]2.0.Co;2

[ece35967-bib-0084] Schill, K. , & Yahner, R. (2009). Nest‐site selection and nest survival of early successional birds in central Pennsylvania. The Wilson Journal of Ornithology, 121(3), 476–484. 10.1676/08-014.1

[ece35967-bib-0085] Schlossberg, S. (2009). Site fidelity of shrubland and forest birds. The Condor, 111(2), 238–246. 10.1525/cond.2009.080087

[ece35967-bib-0086] Schlossberg, S. , & King, D. I. (2007). Ecology and management of scrub‐shrub birds in New England: A comprehensive review. Report submitted to Natural Resources Conservation Service, Resource Inventory and Assessment Division, Beltsville, Maryland, USA.

[ece35967-bib-0089] Schlossberg, S. , King, D. I. , Chandler, R. B. , & Mazzei, B. A. (2010). Regional synthesis of habitat relationships in shrubland birds. The Journal of Wildlife Management, 74, 1513–1522. 10.1111/j.1937-2817.2010.tb01279.x

[ece35967-bib-0090] Seidl, R. , Rammer, W. , & Spies, T. (2014). Disturbance legacies increase the resilience of forest ecosystem structure, composition, and functioning. Ecological Applications, 24(8), 2063–2077. 10.1890/14-0255.1 27053913 PMC4820056

[ece35967-bib-0091] Smith, A. , Koper, N. , Francis, C. , & Fahrig, L. (2009). Confronting collinearity: Comparing methods for disentangling the effects of habitat loss and fragmentation. Landscape Ecology, 24(10), 1271–1285. 10.1007/s10980-009-9383-3

[ece35967-bib-0092] Staudhammer, C. , & LeMay, V. (2001). Introduction and evaluation of possible indices of stand structural diversity. Canadian Journal of Forest Research, 31(7), 1105–1115. 10.1139/x01-033

[ece35967-bib-0093] Stauffer, D. F. , & Best, L. B. (1986). Nest‐site characteristics of open‐nesting birds in riparian habitats in Iowa. The Wilson Bulletin, 98, 231–242.

[ece35967-bib-0095] Taulman, J. F. (2013). A comparison of fixed‐width transects and fixed‐radius point counts for breeding‐bird surveys in a mixed hardwood forest. Southeastern Naturalist, 12(3), 457–477. 10.1656/058.012.0301

[ece35967-bib-0096] Thomas, S. C. , & MacLellan, J. (2004). Boreal and temperate forests. In Owens J. N. & Lund H. G. (Eds.), Forests and forest plants (pp. 152–175). Oxford, UK: Encyclopedia of Life Support Systems (EOLSS); UNESCO, Eolss Publishers.

[ece35967-bib-0097] Thompson, F. R. , & DeGraaf, R. M. (2001). Conservation approaches for woody, early successional communities in the eastern United States. Wildlife Society Bulletin, 29(2), 483–494.

[ece35967-bib-0098] Thompson, W. L. (2002). Towards reliable bird surveys: Accounting for individuals present but not detected. The Auk, 119(1), 18 10.1642/0004-8038(2002)119[0018:trbsaf]2.0.co;2

[ece35967-bib-0099] Tozer, D. , Burke, D. , Nol, E. , & Elliott, K. (2010). Short‐term effects of group‐selection harvesting on breeding birds in a northern hardwood forest. Forest Ecology and Management, 259(8), 1522–1529. 10.1016/j.foreco.2010.01.028

[ece35967-bib-0100] Ulyshen, M. (2011). Arthropod vertical stratification in temperate deciduous forests: Implications for conservation‐oriented management. Forest Ecology and Management, 261(9), 1479–1489. 10.1016/j.foreco.2011.01.033

[ece35967-bib-0101] Vanderwel, M. , Malcolm, J. , & Mills, S. (2007). A Meta‐analysis of bird responses to uniform partial harvesting across North America. Conservation Biology, 21(5), 1230–1240. 10.1111/j.1523-1739.2007.00756.x 17883488

[ece35967-bib-0102] Verschuyl, J. , Hansen, A. , McWethy, D. , Sallabanks, R. , & Hutto, R. (2008). Is the effect of forest structure on bird diversity modified by forest productivity. Ecological Applications, 18(5), 1155–1170. 10.1890/07-0839.1 18686578

[ece35967-bib-0103] Welsh, C. J. , & Healy, W. M. (1993). Effect of even‐aged timber management on bird species diversity and composition in northern hardwoods of New Hampshire. Wildlife Society Bulletin, 21(2), 143–154.

[ece35967-bib-0104] Whelan, C. J. , & Maina, G. G. (2005). Effects of season, understorey vegetation density, habitat edge and tree diameter on patch‐use by bark‐foraging birds. Functional Ecology, 19(3), 529–536. 10.1111/j.1365-2435.2005.00996.x

[ece35967-bib-0105] Winkler, D. (2005). Ecological succession of breeding bird communities in deciduous and coniferous forests in the Sopron Mountains, Hungary. Acta Silvatica Et Lignaria Hungarica, 1, 49–58.

[ece35967-bib-0107] Zenner, E. , Kabrick, J. , Jensen, R. , Peck, J. , & Grabner, J. (2006). Responses of ground flora to a gradient of harvest intensity in the Missouri Ozarks. Forest Ecology and Management, 222(1–3), 326–334. 10.1016/j.foreco.2005.10.027

