# Supplementary material for: Legacy forest structure increases bird diversity and abundance in aging young forests
Source: Ecol Evol. 2020 Jan 28;10(3):1193–208. doi: 10.1002/ece3.5967 (PMC7029076; doi:10.1002/ece3.5967)
Supplement: Supplementary file 1 [file ECE3-10-1193-s001.docx]

**Appendix - S1**

**Table S1**. Species observed in the study classified by nesting functional guild based on information provided by the Cornell Lab of Ornithology.

| **Guild** | **Species** |
| --- | --- |
| Shrub nesting  *These species nest below or within the dense structure of under- or ground-story shrubs.* | Grey Catbird (*Dumetella carolinensis*) |
|  | Yellow Warbler (*Setophaga petechial*) |
|  | Common Yellowthroat (*Geothlypis trichas*) |
|  | Chipping Sparrow (*Spizella passerine*) |
|  | Prairie Warbler (*Setophaga discolor*) |
|  | Chestnut-sided Warbler (Setophaga pensylvanica) |
|  | American Goldfinch (Spinus tristis) |
|  | Northern Cardinal (*Cardinalis cardinalis)* |
|  | Indigo Bunting (*Passerina cyanea*) |
|  | Eastern Towhee (*Pipilo erythrophthalmus*) |
|  | Black-throated blue warbler (*Setophaga caerulescens*) |
|  | Northern mockingbird (*Mimus polyglottos*) |
|  | Swamp sparrow (*Melospiza georgiana*) |
|  | Blue-winged warbler (*Vermivora cyanoptera*)* |
| Forest ground-nesting  *These species nest in the understory of a close-canopied forest, often constructing nests out of leaf litter.* | Canada Warbler (*Cardellina canadensis*) |
|  | Black and White Warbler (*Mniotilta varia*) |
|  | Wild Turkey (*Meleagris gallopavo*) |
|  | Ovenbird (*Seiurus aurocapilla*) |
|  | Veery (*Catharus fuscescens*) |
|  | Worm-eating warbler (*Helmitheros vermivorum*) |
|  | Northern waterthrush (*Parkesia noveboracensis*) |
|  | Ruffed grouse (*Bonasa umbellus*) |
|  | Hermit thrush (*Catharus guttatus*) |
| Cavity-nesting  *These species nest within holes in living or, often, standing dead trees.* | Black-capped chickadee (*Poecile atricapillus*) |
|  | Downy woodpecker (*Picoides pubescens*) |
|  | Hairy woodpecker (*Picoides villosus*) |
|  | Great crested flycatcher (*Myiarchus crinitus*) |
|  | Northern flicker (*Colaptes auratus*) |
|  | Pileated woodpecker (*Dryocopus pileatus*) |
|  | Red-breasted nuthatch (*Sitta canadensis*) |
|  | Red-bellied woodpecker (*Melanerpes carolinus*) |
|  | Tree swallow (*Tachycineta bicolor*) |
|  | Tufted titmouse (*Baeolophus bicolor*) |
|  | White-breasted nuthatch (*Sitta carolinensis*) |
|  | Yellow-bellied sapsucker (*Sphyrapicus varius*) |
|  | American kestrel (*Falco sparverius*) |
|  | Black-vulture (*Coragyps atratus*) |
| Tree-canopy-nesting  *These species nest on living and dead tree branches and boles, at varying heights and locations.* | American crow (*Corvus brachyrhynchos*) |
|  | American redstart (*Setophaga ruticilla*) |
|  | American robin (*Turdus migratorius*) |
|  | Baltimore oriole (*Icterus galbula*) |
|  | Black-billed cuckoo (*Coccyzus erythropthalmus*) |
|  | Brown-headed cowbird (*Molothrus ater*) |
|  | Blue-headed vireo (*Vireo solitarius*) |
|  | Blackburnian warbler (*Setophaga fusca*) |
|  | Blue jay (*Cyanocitta cristata*) |
|  | Brown creeper (*Certhia americana*) |
|  | Black-throated green warbler (*Setophaga virens*) |
|  | Broad-winged hawk (*Buteo platypterus*) |
|  | Cedar waxwing (*Bombycilla cedrorum*) |
|  | Eastern kingbird (*Tyrannus tyrannus*) |
|  | Eastern wood-pewee (*Contopus virens*) |
|  | Golden crowned kinglet (*Regulus satrapa*) |
|  | Least flycatcher (*Empidonax minimus*) |
|  | Magnolia warbler (*Setophaga magnolia*) |
|  | Mourning dove (*Zenaida macroura*) |
|  | Pine warbler (*Setophaga pinus*) |
|  | Rose-breasted grosbeak (*Pheucticus ludovicianus*) |
|  | Red eyed vireo (*Vireo olivaceus*) |
|  | Red-tailed hawk (*Buteo jamaicensis*) |
|  | Ruby-throated hummingbird (*Archilochus colubris*) |
|  | Scarlet tanager (*Piranga olivacea*) |
|  | Wood thrush (*Hylocichla mustelina*) |
|  | Yellow-billed cuckoo (*Coccyzus americanus*) |
|  | Yellow-throated vireo (*Vireo flavifrons*) |
|  | Yellow-rumped warbler (*Setophaga coronata*) |
|  | Canada Goose (*Branta Canadensis*) |
| Marsh/open ground-nesting  *These species nest in large open areas, often near water.* | Red-winged Blackbird (*Agelaius phoeniceus*) |
|  | Northern Harrier (*Circus cyaneus*) |
|  |  |

*The blue-winged warbler was in shrub-nesting category because, though it’s a ground-nester, it nests on the ground in early to mid-successional habitats

**Appendix - S2**

Figure S2. Biplot of results of principal components analysis of vegetation variables, excluding percent canopy cover. Stand phase indicated by color. EI – Early initiation; LI- Late initiation; ESE – Early stem exclusion; UM – Control mature forest


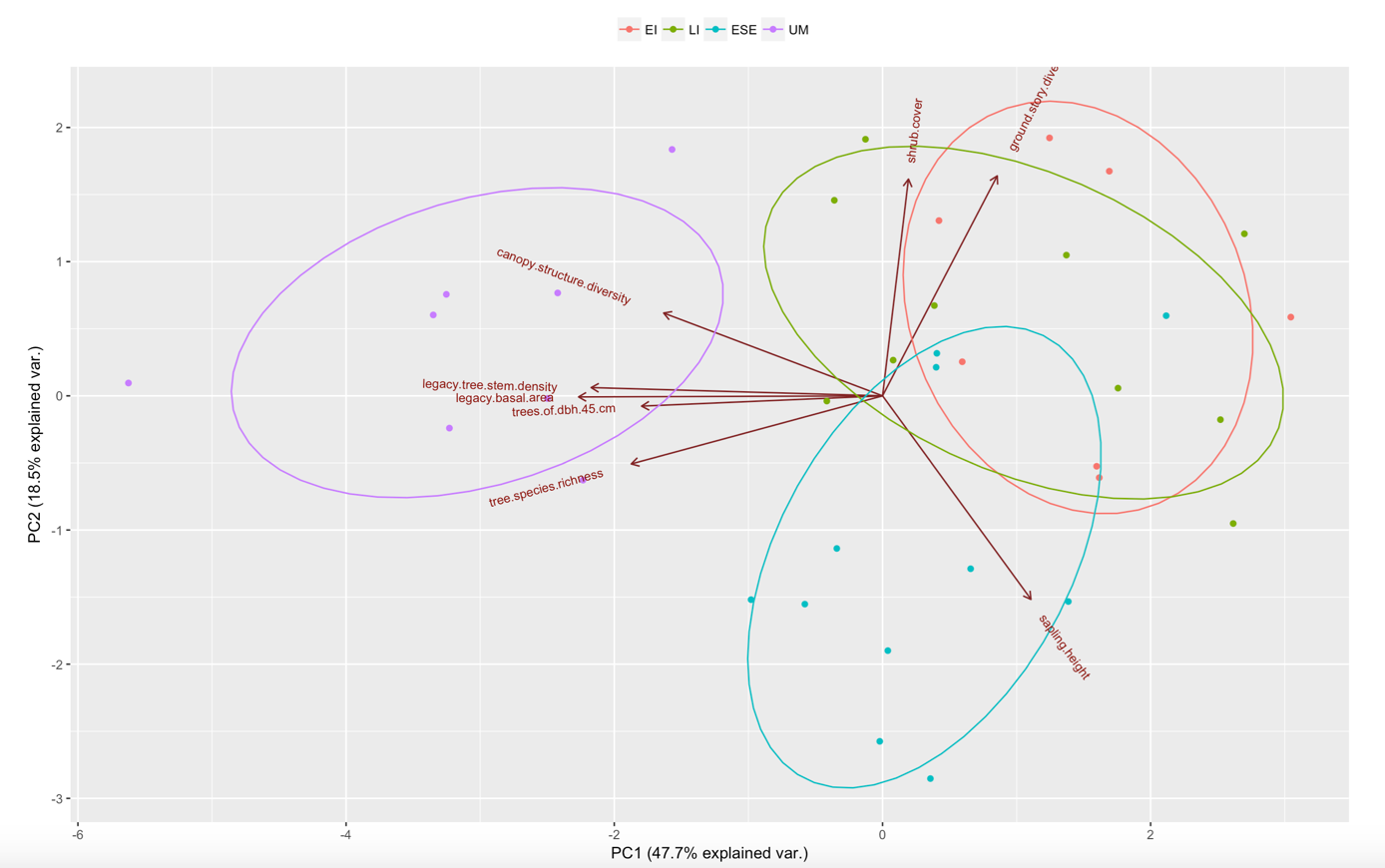


**Appendix - S3**

Table 1 ANOVA or Kruskal-Wallis test of response of vegetation variables to stand phase. ANOVA conducted if data is normally distributed; and if it is not then it is determined by the Kruskal-Wallis test.

Kruskal- Wallis ANOVA

x^2^ df p F Pr>F

Percent canopy cover 15.3 2 SE-04

Sapling median height 8.1 2 0.017

Ground-story plant diversity 8.58 0.001

Total shrub cover 8.9 2 0.012

Stem density of trees with dbh >45 cm 2.4 2 0.306

Legacy basal area 2.27 0.124

Legacy stem density 4.26 0.026

Total tree species richness 6.9 2 0.032

Canopy structure Shannon diversity 0.2 2 0.897

Table 2 ANOVA or Kruskal-Wallis test of response of bird functional guild evenness and species richness to stand phase. ANOVA conducted if data is normally distributed, and if it is not then it is determined by the Kruskal-Wallis test.

Kruskal- Wallis ANOVA

x^2^ df p F Pr>F

Shrub-nesting bird evenness 2.09 0.145

Forest ground-nesting bird evenness 2.46 0.105

Tree-nesting bird evenness 1.56 0.231

Cavity-nesting bird evenness 1.8 2 0.412

Foliage-gleaning bird evenness 0.72 0.495

Ground-foraging bird evenness 2.25 0.126

Shrub-nesting bird richness **6.63 0.005**

Forest ground-nesting bird richness 5.3 2 0.071

Tree-nesting bird richness 2.60 0.094

Cavity-nesting bird richness **5.92 0.008**

Ground-foraging bird richness 0.13 0.875
